# Grin1 Receptor Deletion within CRF Neurons Enhances Fear Memory

**DOI:** 10.1371/journal.pone.0111009

**Published:** 2014-10-23

**Authors:** Georgette Gafford, Aaron M. Jasnow, Kerry J. Ressler

**Affiliations:** 1 Department of Psychiatry and Behavioral Sciences, Yerkes National Primate Research Center, Emory University School of Medicine, Atlanta, Georgia, United States of America; 2 Department of Psychological Sciences, Kent State University, Kent, Ohio, United States of America; Oregon Health and Science University, United States of America

## Abstract

Corticotropin releasing factor (CRF) dysregulation is implicated in mood and anxiety disorders such as posttraumatic stress disorder (PTSD). CRF is expressed in areas engaged in fear and anxiety processing including the central amygdala (CeA). Complicating our ability to study the contribution of CRF-containing neurons to fear and anxiety behavior is the wide variety of cell types in which CRF is expressed. To manipulate specific subpopulations of CRF containing neurons, our lab has developed a mouse with a Cre recombinase gene driven by a CRF promoter (CRFp3.0Cre) (Martin et al., 2010). In these studies, mice that have the gene that encodes NR1 (*Grin1*) flanked by loxP sites (floxed) were crossed with our previously developed CRFp3.0Cre mouse to selectively disrupt *Grin1* within CRF containing neurons (Cre+*/^fGrin1+^*). We find that disruption of *Grin1* in CRF neurons did not affect baseline levels of anxiety, locomotion, pain sensitivity or exploration of a novel object. However, baseline expression of *Grin1* was decreased in Cre+*/^fGrin1+^* mice as measured by RTPCR. Cre+*/^fGrin1+^* mice showed enhanced auditory fear acquisition and retention without showing any significant effect on fear extinction. We measured *Gria1*, the gene that encodes AMPAR1 and the CREB activator Creb1 in the amygdala of Cre+/*^fGrin1+^* mice after fear conditioning. Both *Gria1* and *Creb1* were enhanced in the amygdala after training. To determine if the *Grin1*-expressing CRF neurons within the CeA are responsible for the enhancement of fear memory in adults, we infused a lentivirus with Cre driven by a CRF promoter *(*LV pCRF-Cre*/^fGrin1+^*) into the CeA of floxed *Grin1* mice. Cre driven deletion of *Grin1* specifically within CRF expressing cells in the CeA also resulted in enhanced fear memory acquisition and retention. Altogether, these findings suggest that selective disruption of *Grin1* within CeA CRF neurons strongly enhances fear memory.

## Introduction

Fear-related disorders such as posttraumatic stress disorder (PTSD) are marked by enhanced fear memory and resistance to fear extinction [1]–[4]. Pavlovian fear conditioning and fear extinction provide ideal tools to model fear memory processes and define new treatments for anxiety disorders such as PTSD. Pavlovian fear conditioning occurs when a novel cue (Conditioned Stimulus, CS) is paired with an aversive event (Unconditioned Stimulus, US) and results in increased fear behavior (the Conditioned Response, CR). Fear extinction is an inhibitory learning process where the CS is presented alone without the US, resulting in a gradual decrease in the conditioned fear response (CR).

The amygdala is a critical node in the processing of acquisition, consolidation and extinction of fear memory. The central amygdala (CeA), a subnuclei of the amygdala has been shown to be crucial for fear learning [5]. The CeA includes a population of corticotropin-releasing factor (CRF) peptide containing neurons [6]. This population of CRF neurons in the CeA is of interest because they have been implicated in learning and memory [7], and are activated in response to a variety of stressors [8]–[10]. In humans, high levels of CRF have been found in the cerebrospinal fluid of those diagnosed with PTSD, with the highest CRF levels correlated with the greatest symptom severity [11], [12].

NMDARs (N-methyl-D-aspartate receptors) are also expressed in neurons in the CeA [13]–[16] and have been shown to be engaged during synaptic plasticity [17] and fear conditioning [18], [19]. In general, relative levels of AMPA and NMDA receptors determine memory strength [20]–[22] and drugs that enhance memory increase the levels of these receptors [23]. Activation of NMDARs requires membrane depolarization through AMPARs (2-amino-3-5-methyl-3-oxo-1, 2-oxazol-4-yl propanoic acid receptor) that lead to the production of transcription factors (i.e. CREB) and insertion of new AMPARs into the membrane [23]. Though it is broadly known that CeA NMDARs are important for fear memory, little is known about how NMDARs modulate different cell types within the CeA.

Published work points to a functional relationship between the NMDAR and CRF containing neurons. For example, one recent study showed that many NR1 (NMDAR1) containing somata and dendrites in the CeA coexpress CRF [16]. A separate study showed that in vitro CRF application resulted in an NMDA receptor dependent long-term potentiation of amygdala inputs to the CeA (Pollandt et al., 2006). Further, in vivo administration of CRF into the CeA increases presynaptic glutamate release after a stressor [24]. These experiments indicate that CRF modulates activation of NMDAR containing neurons.

The present work set out to determine specifically how CRF containing NR1 neurons contribute to fear memory formation and extinction. Mice that have the gene that encodes NR1 (*Grin1,* Glutamate receptor, ionotropic, N-methyl D-aspartate 1) flanked by loxP sites (floxed) were crossed with our previously developed CRFp3.0Cre mouse [25] resulting in a CRF neuron-specific deletion of the *Grin1* gene (Cre+/^f*Grin1*+^). We find disruption of *Grin1* in CRF containing neurons enhances fear memory acquisition and retention without effecting baseline measures of anxiety. Further, since AMPAR and CREB expression have been linked to changes in NMDAR expression [23], we measured levels of the genes for AMPAR1 (*Gria1*, glutamate receptor, ionotropic, AMPA 1) and the CREB activator CREB1 (*Creb1,* cAMP response element-binding protein 1) in the amygdala. We found increased expression of *Gria1* and *Creb1* in the amygdala after training in Cre+/^f*Grin1*+^ mice compared to littermate controls. A final experiment determined that virally-directed deletion of *Grin1* restricted to CRF containing CeA neurons also enhanced fear acquisition and retention. This suggests that the effects underlying the enhancement in fear memory are occurring within the CeA.

Together, these findings highlight a cell type-specific behavioral profile for *Crf*-*Grin1* containing neurons in the CeA, in which disruption of glutamatergic regulation within a subpopulation of CRF containing neurons enhances fear memory.

## Materials and Methods

### Production of Transgenic Mice

All experiments were performed on adult (6–10 weeks old) male mice bred within our laboratory. Animal procedures were approved by the Institutional Animal Care and Use Committee of Emory University (Atlanta, GA) and were in compliance with National Institutes of Health guidelines. CRFp3.0Cre+ (CRF Cre+, RRID:IMSR_ 011087) transgenic mice [25], [26] were crossed with *Grin1* floxed (f*Grin1*) mice in a C57/FVB background (Jackson Labs Strain: B6.129S4-Grin1 tm2Stl/J; RRID:IMSR_JAX:005246) [27]. Mice were back-crossed until offspring were either positive or negative for Cre and all mice were homozygous for f*Grin1* resulting in Cre+/^f*Grin1*+^ or Cre−/^f*Grin1*+^ mice. Cre−/^f*Grin1*+^ littermates of Cre+/^f*Grin1*+^ were used as controls in all experiments. In the presence of Cre recombinase the transmembrane domain of the *Grin1* gene is deleted. DNA from transgenic mice was assessed for expression of Cre (5′ GCATTAC CGGTCGATGCAACGAGTGATGAG; 3′GAGTGAACGAACCTGGTCGAAATCAG TGCG) as well as expression of ‘floxed’ *Grin1* (reverse 5′ GTGCTGGGATCCACATTCAT 3′; forward 5′ AAACAGGGCTCAG TGGGTAA 3′). Cre and NMDAR1 expression were determined using the following thermal cycling programs: Cre: Stage 1, 93C (1 minute); Stage 2 (25 cycles of 93C, 20 seconds and 68 C, 3 minutes). NR1: Stage 1, 94C (3 minutes); Stage 2 (35 cycles of 94C, 30 seconds, 61 C,1 minute, 72C, 1 minute) Stage 3, 72C (2 minutes).

### Lentivirus Production and Infusions

Lentiviral production was performed by the Emory Viral Vector Core (http://neurology.emory.edu/ENNCF/viral_vector/). Following previous protocols [28]–[30] using the CRF promoter to drive Cre (LV-pCRF_3.0_-Cre) [25] or a CRF driven green fluorescent protein (GFP)-expressing control vector (LV-pCRF-GFP), delta8.9 and VSV-g packaging and capsid constructs were co-transfected into HEK293T producer cells to produce replication incompetent but highly infective virus. The packaged virus was concentrated through a number of ultracentrifugation steps and titered to reach at least 1×10^9^ IU/ml. LV-pCRF-GFP -expressing control vector or LV-pCRF_3.0_-Cre -recombinase expressing vector virus was bilaterally infused using a 26-gauge Hamilton syringe (precoated with bovine serum albumin) with a microinjection pump (0.25 µl/15 min) into the CeA (From Bregma: AP −1.3; ML+/−2.6; DV-4.4) of homozygous f*Grin1* mice using stereotaxic surgery under ketamine (75 mg/kg)/dormitor (1 mg/kg) anesthesia. Diffusion was allowed for 10 minutes and the syringe was then slowly retracted. Animals received sutures to close the wound and Antisedan (1 mg/kg) to reverse the effects of Ketamine. Metacam (1 mg/kg) was administered for pain during surgery and for the 3 days following surgery.

### LacZ Staining

Staining for LacZ was done as previously reported [25]. In short, CRFp3.0Cre transgenic mice were crossed with a strain containing a floxed stop-LacZ construct in the Rosa26 locus (Rosa LacZ, Jackson Laboratories). Cryostat sectioned slices from CRFp3.0Cre-LacZ offspring were rinsed in phosphate-buffered saline then incubated overnight in X-Gal solution.

### *In Situ* hybridization

For *In situ* hybridization brains were rapidly removed following a lethal dose of anesthesia, sectioned at 20 µM, and placed onto SuperFrost Plus slides and stored at −80°C until further processing [26]. The 35S-UTP labeled riboprobes were prepared from linearized clones and purified. The probe was diluted in hybridization buffer (50% (vol/vol) deionized formamide, 10 mm DTT, 20 mm Tris, 300 mm sodium chloride, 5 mm EDTA, 10% (vol/vol) dextran sulfate, 1% Denhardt’s solution, 0.5 mg/mL yeast RNA, and 10 mm NaH2PO4). Sections were incubated overnight in humid chambers at 50°C. Following hybridization, slides were put through stringent SSC washes and dehydrated with increasing concentrations of ethanol. Slides were air dried and exposed to Biomax film.

### Quantitative RTPCR

RNA from amygdala and BNST of auditory fear conditioned *Cre*−*/^fGrin1 or^ Cre+/^fGrin1^* mice was extracted one hour after training. In brief, the tissue was homogenized and centrifuged at 13,000×g for 3 min. RNA was washed with 70% ETOH and purified using RNeasy columns (Qiagen). The quantity and amount of RNA was determined using a NanoDrop spectrophotometer. RNA (180 ng) from the amygdala and BNST were reverse transcribed into cDNA using the RT2-First Strand Kit (C-03, SA Biosciences). Quantitative PCR was performed using the Applied Biosystems 7500 Fast System using the following thermal cycling program: Stage 1, 95 C (10 minutes); Stage 2, (40 cycles of 95 C, 15 seconds and 60 C, 1 minute). Data collection occurred during the 60C 1 minute step of Stage 2. SYBR Green Taq polymerase was used with primers for *Creb1*, *Grin1*, *CRF*, *Gria1* and *Gapdh* (SA Biosciences: *Creb1*, PPM03382F; *Grin1*, PPM04235A; *CRF*, PPM04632A; *Gria1*, PPM04285C; *Gapdh,* PPM02946E). All genes of interest were normalized to their individual *Gapdh* levels and then normalized to the averaged *Cre*−*/^fGrin1^*control.

### Dual FISH (Fluorescent *In Situ* Hybridization)

Sections were prepared for fluorescent in situ hybridization using the same probes as those from the *in situ* hybridization experiments, with the exception that FITC (*CRF*) and DIG (*Grin1*), RNA labeling mix (Roche) was used instead of S^35^ as previously described [26]. Slides were hybridized at 65°C for at least 12 h, followed by a series of stringent washes. Sections were then blocked with 1% TNB buffer (1% BSA in TN) for 30 min, treated with peroxidase followed by anti-DIG antibody (1:500 dilution; Roche) for 1.5 h. Sections were rinsed and treated with Cy3 antibody (1:50 dilution; Roche) for 30 min in the dark. After further rinses, sections were similarly treated with anti FITC antibody (1:500 dilution; Vector Laboratories) followed by FITC tyramide (1:50) for amplification in a humid chamber in the dark. Finally, sections were stained with Hoescht stain and cover-slipped with Vectashield (Vector Laboratories).

### Shock Reactivity

Shock reactivity data was recorded using the SR-LAB startle response system (San Diego Instruments) and conducted as previously described [26]. An accelerometer recorded the amplitude of chamber displacement with shock during the fear conditioning session. The average amplitude of accelerometer displacement in response to shock was recorded and compared across groups.

### Open Field Test

The open field test was conducted using MED PC boxes and consisted of an arena in which the central zone was 6 cm from the perimeter of the chamber walls. Activity was monitored using 24-beam infrared arrays across the base of each chamber wall (MED Associates, model OFA-MS). Activity data was collected and analyzed with the MED Associates Activity Monitor Data Analysis software. Over the ten minute test session, multiple measures were recorded with a 50 ms resolution including distance traveled and time in center and surround.

### Elevated Plus Maze

The elevated plus maze consisted of four arms (30×5 cm) elevated 50 cm from the floor. The room was dimly lit to encourage exploration and each animal was placed into the center area of the elevated plus maze facing a closed arm. Over a 5 minute period, mice freely explored the maze. The number of open arm entries and distance traveled was recorded and verified by an observer blind to group condition.

### Novel Object Exploration

To test for differences in baseline novelty behavior, mice were exposed to two novel objects for 10 minutes each for two days. On the third day mice were presented again with the familiar object however the second object was replaced with a completely novel object. Time spent exploring each object was calculated using Limelight software (Coulbourn Instruments) and verified by an observer blind to group condition.

### Auditory Fear Conditioning and Extinction

Animals were tested for cue dependent fear conditioning and extinction. All groups received 5 CS tones (30s, 6 kHz, 74dB) co-terminating with US shocks (500 ms, 1 mA) with a 90 second inter trial interval (ITI) [26], [31]. Average freezing behavior from each of the 30 second tone – shock trials during training was compared across groups. During extinction training and testing, mice received 15 or 30 CS tones (30s, 6 kHz, 74dB, 90 second ITI) in a novel context that differed in size, shape, olfactory cue and lighting from the training context. Fear conditioning and extinction were conducted in Med Associates fear conditioning chambers. Actimetrics video-based Freezeframe version 3 software was used to detect and analyze freezing behavior from video.

### Statistical Analysis

Baseline freezing behavior prior to fear conditioning and extinction was averaged across the 180 second baseline and compared using Oneway ANOVA. Repeated measures ANOVA was used to test for differences in freezing during the tone – shock presentation of the fear conditioning session. During extinction training and testing, freezing behavior across 3 CS presentations was averaged. These averaged points were compared using repeated measures ANOVA. Oneway ANOVA was used to analyze data RTPCR data. Fold change relative to control was calculated by determining the difference in the number of cycles it takes to reach threshold between the target gene and housekeeping gene (*Gapdh*). Resulting values were compared using Oneway ANOVA.

## Results

### LacZ Staining

**Figure 1:**
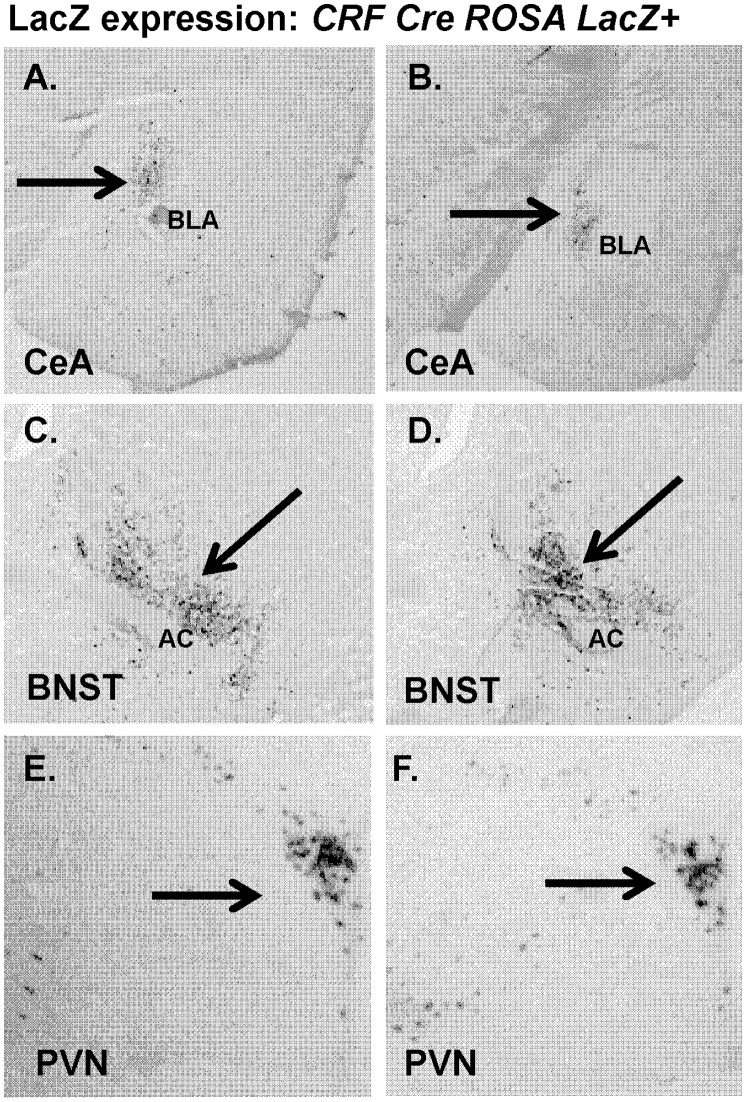
LacZ expression is specific to CRF enriched areas in *CRF-Cre ROSA LacZ+* mice. Images showing LacZ expression in (A, B) central amygdala (CeA). (C, D) bed nucleus of stria terminalis (BNST),and (E, F) paraventricular nucleus (PVN) of *CRF-Cre ROSA LacZ+* mice.

As in previous work [25] we assessed LacZ expression in CRF Cre mice crossed with Rosa LacZ mice. Visualization of sections from CRF Cre- LacZ mice (N = 4) showed staining localized to CeA (Figure 1 A, B), BNST (Figure 1C, D) and PVN (Figure 1E, F), in agreement with localization of CRF expression.

### *In Situ* Hybridization

**Figure 2:**
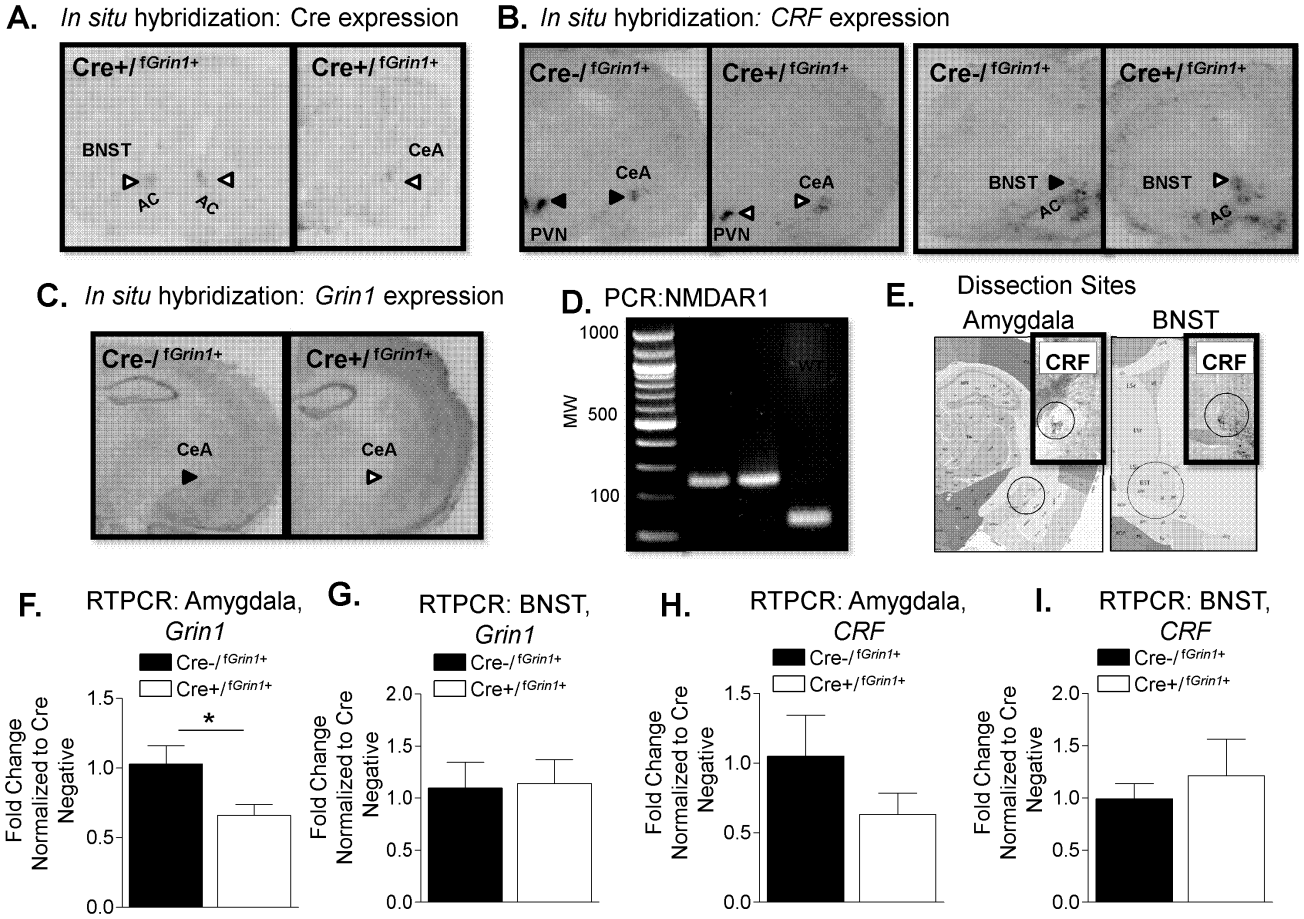
In situ hybridization conducted on sections from (A) Cre+/^f^ ^*Grin1*+^ mice show Cre expression in CRF containing areas (*Left,* BNST; *Right,* CeA). Representative expression of (B) *CRF* in Cre−/^f*Grin1*+^ (*Left*, black arrowheads) and Cre+/^f*Grin1*+^ (*Right*, white arrowheads) PVN, CeA and BNST are indicated. Anterior commissure (AC) is indicated for orientation purposes. (C) *Grin1* in Cre−/^f*Grin1*+^ (*Left*, black arrow) and Cre+/^f*Grin1*+^ (*Right*, white arrow) mouse is shown. (D) Image of PCR from Cre+/^f*Grin1*+^ or Cre−/^f*Grin1*+^ mice (280 bp) compared to wildtype (WT, 180 bp) mouse. E. Detailed depiction of central amygdala and BNST dissection sites. Inset shows CRF expression images from CRF-Cre ROSA LacZ+ mice to indicate overlap of punch area with CRF expression**.** F. RTPCR shows a significant difference in baseline expression of Grin1 in the amygdala, but not in the (G) BNST in Cre+/^f*Grin1*+^ mice compared to Cre−/^f*Grin1*+^ controls. (H) RTPCR conducted on tissue from the amygdala or from the BNST showed no significant difference in *CRF* expression (I) *Grin1* in Cre+/^f*Grin1*+^ mice compared to Cre−/^f*Grin1*+^ controls.

In situ hybridization was conducted to identify the pattern of Cre expression in Cre+/^f*Grin1*+^ mice. We found Cre to be localized to CRF enriched areas (representative images shown in Figure 2A). In situ hybridization of sections from Cre+/^f*Grin1*+^ and Cre−/^f*Grin1*+^ mice (N = 4 per group) showed representative expression of *CRF* (Figure 2B) and *Grin1* (Figure 2C).

### Baseline RTPCR

We measured *Grin1* and *CRF* mRNA expression using RTPCR in the amygdala (Cre−/^f*Grin1*+^, N = 5; Cre+/^f*Grin1*+^, N = 6). We found a significant decrease in *Grin1* expression (Figure 2F) in the amygdala of Cre+/^f*Grin1*+^ mice (F (1,10) = 7.371, p<0.05) compared to Cre−/^f*Grin1*+^. Measurement of baseline *CRF* expression in the amygdala (Figure 2H) showed no statistically significant difference between the groups (F (1,10) = 2.845, p>0.05), however *CRF* expression does trend lower compared to control, which may indicate some compensation by *CRF* when *Grin1*is disrupted. We also measured *CRF* and *Grin1* at baseline in the BNST, another CRF rich area, to determine whether these targets were changed in Cre+/^f*Grin1*+^ mice compared to control. No significant changes were found in either *CRF* (F (1,10) = 0.281, p>0.05) or *Grin1* (F (1,10) <0.019, p>0.05) in the BNST (Figure 2G,I). Raw data for the RTPCR experiments is included within Data S1.

### Tests of Anxiety-Like Behavior

**Figure 3:**
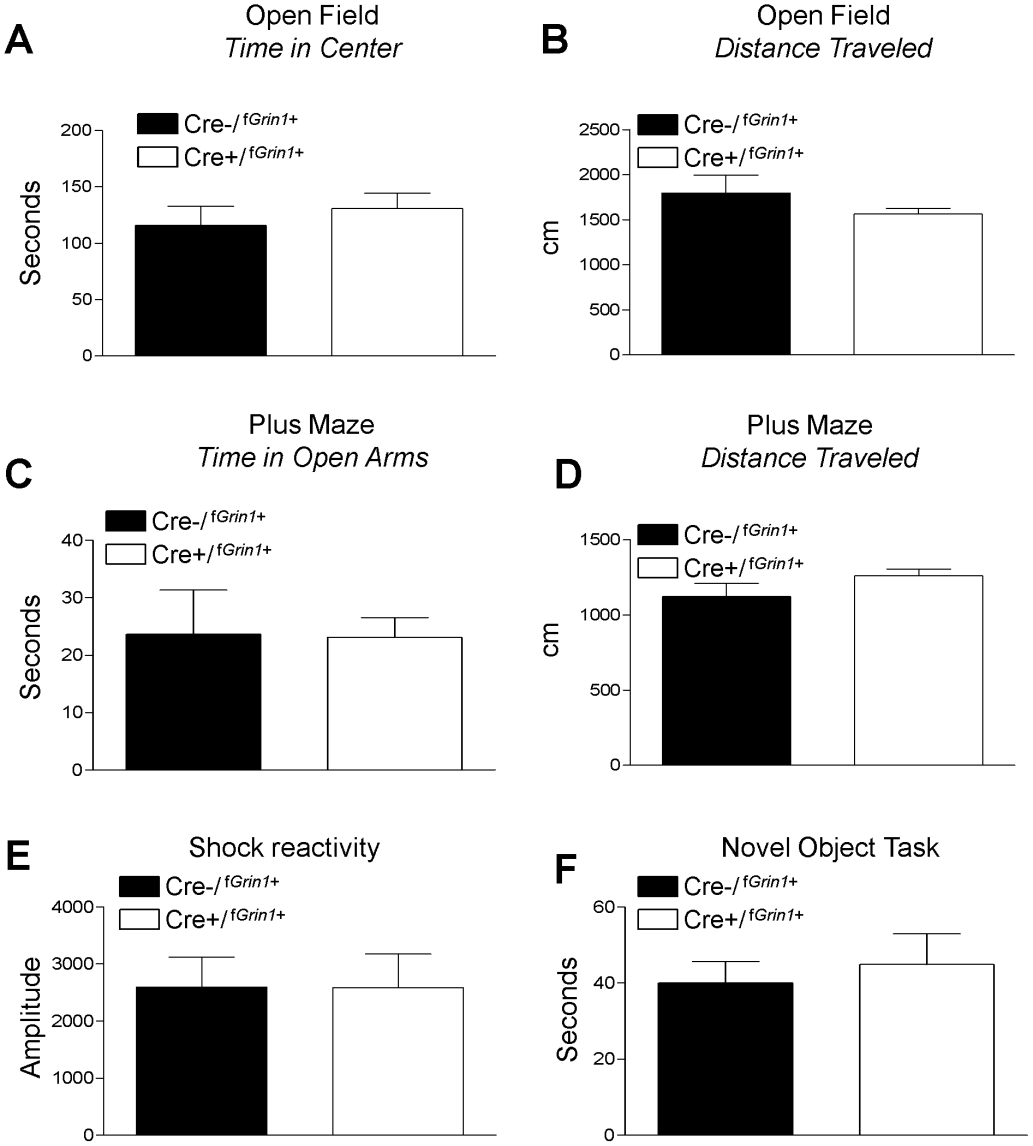
*Grin1* deletion in CRF-containing neurons does not affect locomotion, anxiety-like behavior, or pain reactivity. No significant differences were found between Cre−/^f*Grin1*+^and Cre+/^f*Grin1*+^ mice in anxiety or locomotion as shown by (A) open field time spent in center and (B) open field distance travelled. (C) Time spent in the open arms was not significantly different between Cre−/^f*Grin1*+^and Cre+/^f*Grin1*+^ mice in a plus maze test of anxiety. D. Distance traveled was also not significantly different in the plus maze test. No difference in (E) shock reactivity was found, indicating that mice showed no difference in sensitivity to pain. No differences in activity were seen between Cre−/^f*Grin1*+^and Cre+/^f*Grin1*+^ mice in (F) the novel object task, indicating no difference in novelty seeking behavior.

Cre−/^f*Grin1*+^, (N = 10) and Cre+/^f*Grin1*+^, (N = 12) animals were compared in an open field test. No significant difference was found in time spent (seconds) in the center zone of an open field (F (1, 21) = .48, p>0.05) or distance traveled (centimeters) in the open area of the arena (F (1, 21) = 1.49, p>0.05) indicating no difference in anxiety or general activity level (Figure 3A, B). A separate cohort of Cre−/^f*Grin1*+^, (N = 8) and Cre+/^f*Grin1*+^, (N = 12) mice were tested in the elevated plus maze and no significant difference was found between groups on time spent (seconds) in the open arm (Figure 3C) of the maze (F (1,19) = .005, p>0.05) nor was there a difference in distance (centimeters) traveled (F(1,19) = 2.367, p>0.05, Figure 3D). The raw data for these tests are included in Data S1.

### Shock Reactivity

Mice (Cre−/^f*Grin1*+^, N = 7, Cre+/^f*Grin1*+^, N = 9) were tested for shock reactivity to determine if disruption of *CRF* containing *Grin1* neurons resulted in differences in sensitivity to shock (Figure 3E). No significant differences were found (F (1, 15) = .001, p>0.05). The raw data for this test is included in Data S1.

### Novel Object Exploration

Mice (Cre−/^f*Grin1*+,^ N = 12, Cre+/^f*Grin1*+^, N = 11) were tested for differences in exploration of a novel object (Figure 3F). No significant differences were found (F (1, 22) = .024, p>0.05) in time exploring the novel object.

### Auditory Fear Conditioning and Extinction

**Figure 4:**
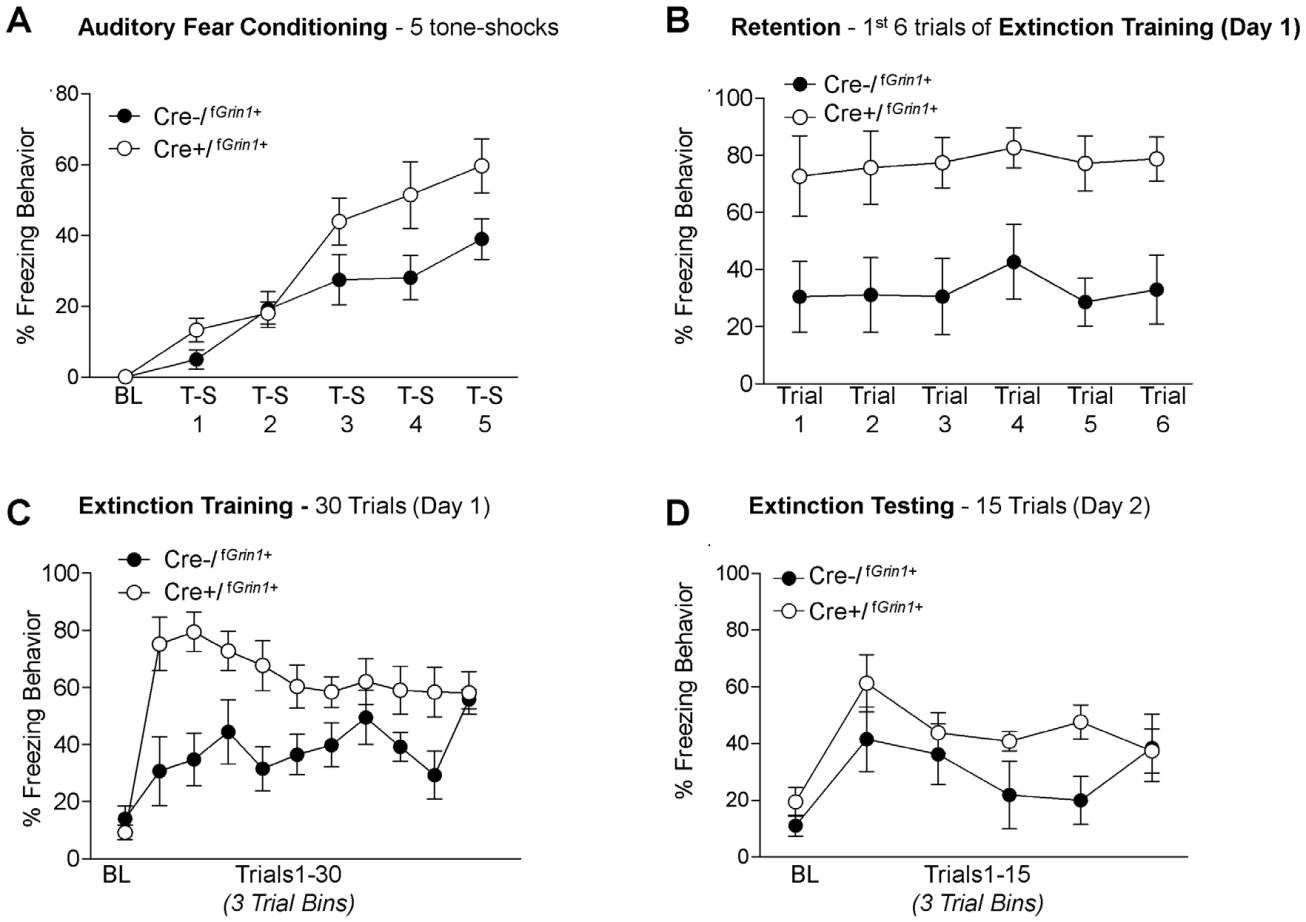
Cre+/^f^ ^*Grin1*+^ mice show enhanced fear memory consolidation and retention. (**A**) Percent freezing during auditory fear conditioning over tone- shock pairings and (**B**) Cre+/^f*Grin1*+^ mice shows significantly more freezing during the 1^st^ six trials of fear extinction (also considered the fear retention test) suggesting an increase in fear memory consolidation. **C**) Average freezing behavior over the entire fear extinction session averaged into bins of 3 trials shows significantly disrupted fear extinction over the 30 extinction trials (**D**) during the extinction test conducted the following day Cre+/^f*Grin1*+^ mice do not show a significant difference from controls.

Cre−/^f*Grin1*+^, (N = 5) or Cre+/^f*Grin1*+^, (N = 5) mice were trained in auditory fear conditioning using 5 tone shock trials. No significant difference was found using Oneway ANOVA on baseline freezing data prior to training (F (1, 9) = 1.898, p>0.05). A repeated measures ANOVA of the 5 tone – shock pairings showed a significant effect of Trial (F (4, 32) = 29.9, p<0.05) and no significant Trial by Group effect (F (4, 32) = 2.11, p = 0.06). There was a significant between-subjects effect for Group (F(1,8) = 15.159, p<0.05) driven by Cre+/^f*Grin1*+^ mice freezing significantly more than Cre−/^f*Grin1*+^ mice (Figure 4A)**.**

As shown in Figure 4C, no significant difference was found on average baseline freezing data prior to tone CS presentation before extinction training (F (1, 9) = .917, p>0.05). A repeated measures ANOVA comparing the first 6 trials of fear extinction training (fear retention, Figure 4B) showed no main effect of Trial (F (5, 40) = .520, p>0.05) or Trial by Group (F(5, 40) = .080, p>0.05); however, there was a robust significant between-subjects Group effect (F(1,8) = 13.0, p<0.05). These data indicate that during the fear retention test, Cre+/^f*Grin1*+^ mice show significantly enhanced fear. The entire 30 trials of extinction training were averaged into bins of 3 trials and those bins were analyzed using repeated measures ANOVA (Figure 4C). There was no within-subjects effect of Trial (F (9, 72) = 1.16, p>0.05); however, there was a Trial by Group interaction (F(9,72) = 2.28, p<0.05) and a significant between subjects Group effect (F (1,8) = 11.018, p<0.05), with Cre+/^f*Grin1*+^ mice showing more freezing behavior throughout fear extinction training (Figure 4C).

Twenty-four hours later mice were tested for fear extinction retention (Figure 4D). No significant difference was found using Oneway ANOVA on average baseline freezing data prior to tone presentation in the extinction test session (F(1, 9) = 1.812, p>0.05). Data from the 15 trial fear extinction test was averaged into 3 trial bins and a repeated measures ANOVA was conducted. We found a significant effect of Trial (F(4, 32) = 3.06, p<0.05), but no Trial by Group interaction (F(4,32) = 1.62, p>0.05) and no between subjects Group effect (F(1,8) = 1.94, p>0.05) indicating that with sufficient extinction training, Cre+/^f*Grin1*+^ animals were able to eventually extinguish fear to an equivalent level compared to controls. The raw data for these experiments may be found in Data S1.

### Post Acquisition RTPCR

**Figure 5:**
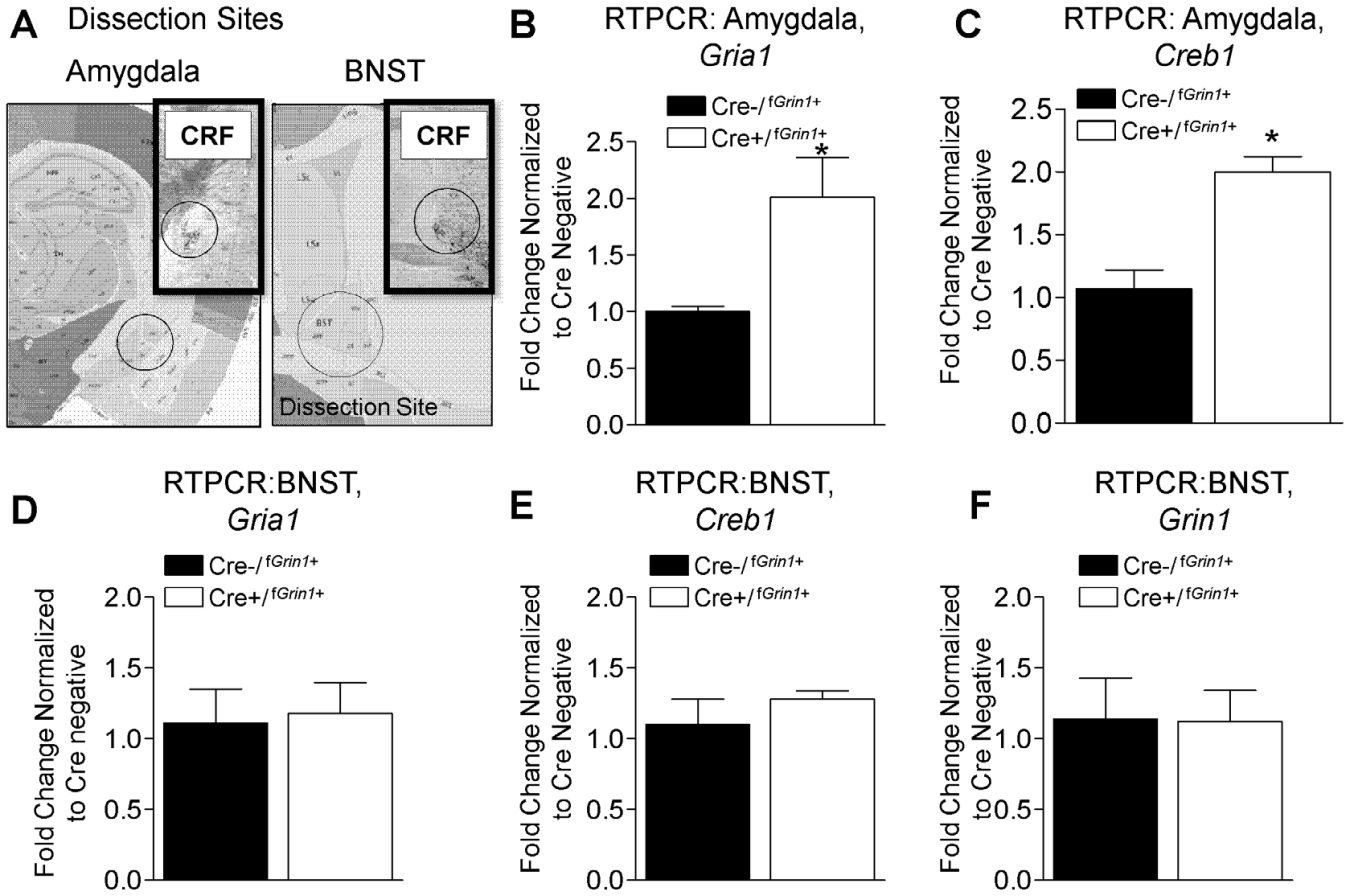
Results from RTPCR of amygdala or BNST after fear conditioning. (**A**) Shows a detailed depiction of dissection sites in central amygdala and BNST. The inset shows CRF expression image the amygdala of CRF-Cre ROSA LacZ+ mice to indicate overlap of the sampling area with CRF expression**.** Cre+/^f*Grin1*+^ mice show a significant increase in (B) *Gria1* and (C) *Creb1* in the amygdala after fear conditioning compared to fear conditioned Cre−/^f*Grin1*+^. No difference between these groups is seen when (D) *Gria1*, (E) *Creb1* or (F) *Grin1* are measured in the BNST after fear conditioning. Inset shows CRF expression images from CRF-Cre ROSA LacZ+ mice in the respective area to indicate overlap of punch with CRF expression**.**

Since acquisition behavior is increased in Cre+/^f*Grin1*+^ mice, we measured targets implicated in strengthening of memory (*Creb1* and *Gria1*) 30 minutes after fear conditioning in the amygdala normalizing Cre+/^f*Grin1*+^ (N = 5) to Cre−/^f*Grin1*+^ (N = 5) control mice. *Gria1* (F (1,9) = 8.079, p<0.05) and *Creb1* (F (1,9) = 23.810, p<0.05) were significantly increased after fear conditioning in the amygdala (Figure 5 B, C). We also measured *Creb1*, *Gria1*and *Grin1* in the BNST as a control structure that strongly expresses CRF. We found that neither *Creb1* (F (1,9) = .881, p>0.05), *Gria1* (F (1,9) = .053, p>0.05), nor *Grin1* (F (1,9) = .004, p>0.05) were significantly different compared to Cre negative controls (Figure 5 D,E,F). These findings indicate the increases in synaptic plasticity related genes, which may be altered in a compensatory fashion secondary to NMDA knockdown in Cre+/^f*Grin1*+^ mice, were not identified in a separate structure enriched in CRF containing neurons (BNST). Raw data for these RTPCR experiments can be found in Data S1.

### CeA-Targeted Infusion of Virus Against CRF-Cre

**Figure 6:**
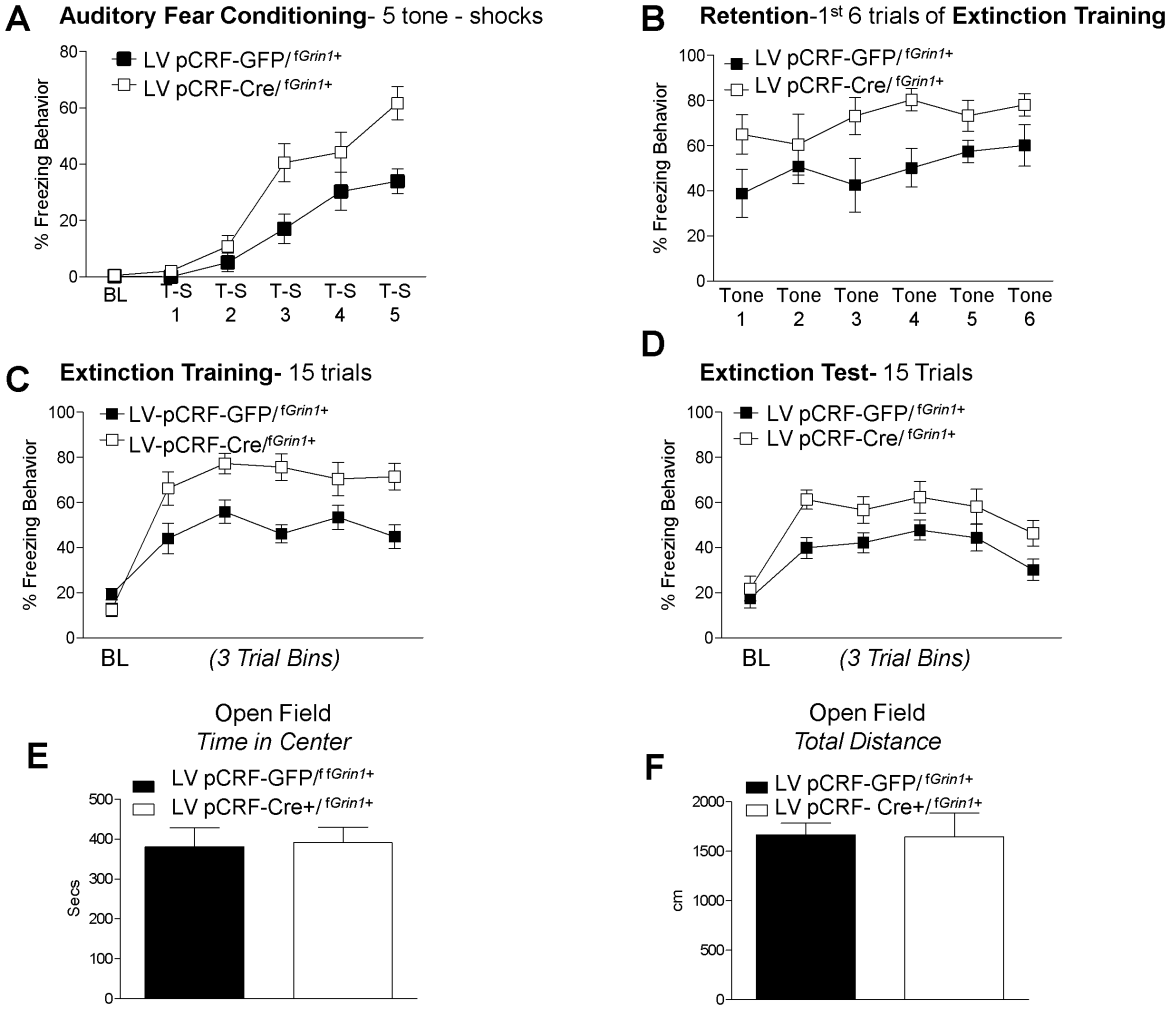
Directed virus against CRF infused into the amygdala of ‘floxed’ *Grin1* mice resulted in enhanced fear memory and delayed fear extinction. Infusion of CRF driven Cre virus into the central amygdala of floxed *Grin1* mice (LV pCRF-Cre/^f*Grin1*+^) significantly (**A**) enhanced auditory fear conditioning compared to those that received control virus (LV-pCRF-GFP/^f*Grin1*+^) and (**B**) retention (1^st^ 5 trials of extinction) tested the following day. A significant disruption was found during (**C**) the extinction training session as well as during (**D**) extinction testing. (**E**) Tests of anxiety-like behavior using the open field test following extinction showed no differences in anxiety behavior in the (**F**) time in center or (**G**) total distance traveled in virus infused mice.

CRF is expressed in neurons across numerous brain regions, including CeA, BNST, hypothalamus, and to a lesser extent, cortex and hippocampus. We next investigated whether manipulating *Grin1* within CRF-specific neurons limited only to the CeA directly enhanced fear acquisition. We infused CRF driven Cre-recombinase expressing vector (LV-pCRF-Cre/^f*Grin1*+^, N = 8) or CRF driven green fluorescent protein -expressing control vector (LV-pCRF-GFP/^f*Grin1*+^, N = 8) into the CeA of f*Grin1* mice. After allowing two weeks for appropriate transgene expression, mice were fear conditioned. Analysis using Oneway ANOVA showed no significant difference between the groups in averaged baseline freezing data prior to training (F (1, 15) = 0.786, p>0.05). A repeated measures ANOVA showed a significant within-subjects effect during training (Figure 6A, F (4, 56) = 2.72, p<0.05) and an overall difference between groups (F (1, 14) = 12.20, p<0.05). This finding indicates that mice with lentivirus-mediated disruption of glutamatergic signaling in CRF containing neurons in the CeA acquire fear conditioning more quickly.

Prior to extinction training there was no significant difference in baseline freezing (F (1, 15) = 3.74, p>0.05). A repeated measures ANOVA conducted on the first 6 trials of extinction training (the fear retention test, Figure 6B) showed no significant Trial (F(1, 14) = 1.296, p>0.05) or Trial x Group interaction (F(1, 14) = .525, p<0.05); however, there was a significant between subjects Group effect showing that LV-pCRF-Cre/^f*Grin1*+^ mice froze significantly more than controls (F(1, 14) = 12.519, p<0.05) and LV-pCRF-Cre/^f*Grin1*+^ mice retain the fear memory better than controls. A repeated measures ANOVA on the entire 15 trials of extinction training (Figure 6C) showed a group effect whereby freezing is significantly higher for LV-pCRF-Cre/^f*Grin1*+^ mice compared to LV-pCRF-GFP/^f*Grin1*+^ controls (F(1, 14) = 17.08, p<0.05). The extinction training protocol for this experiment was 15 trials rather than the more robust extinction protocol used with the transgenic knockdown of *Grin1*– CRF containing neurons. We decreased the number of trials in extinction training to determine whether a significant disruption would be found at extinction test with a less robust extinction training protocol.

No significant difference was found using Oneway ANOVA on baseline freezing data prior to tone presentation during extinction testing (F (1, 15) = 0.384, p>0.05). During the extinction test the following day, repeated measures ANOVA on binned trials of the extinction test showed a significant within subjects effect of Trial (F (4, 56) = 3.436, p<0.05) with no Trial by Group interaction (F (4, 56) = .203, p>0.05). There was a significant difference between subjects effect of Group (Figure 6D, F (1, 14) = 11.57, p<0.05). Raw freezing data for these experiments can be found within Data S1.

**Figure 7:**
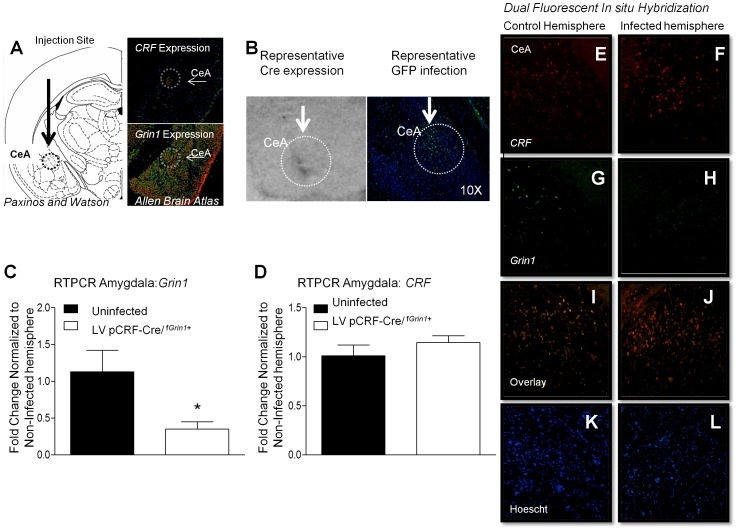
Directed virus against CRF infused into the amygdala of ‘floxed’ *Grin1* mice significantly disrupts *GRIN1* with no significant effects on CRF. (**A**) Intended infusion site of CRF driven Cre virus into the central amygdala of floxed *Grin1* mice (LV pCRF-Cre/^f*Grin1*+^) is illustrated on the *Left* [*53*]. The *upper right* panel shows *CRF* expression in the CeA, *lower right* panel shows *Grin1* expression levels in the CeA. *Image credit*: Allen Institute for Brain Science [54]. (**B**) (*Left*) Representative Cre *in situ* shows representative Cre expression in the CeA of a LV pCRF-Cre/^f*Grin1*+^ infused mouse. *(Right)* Representative GFP infection in the CeA of LV pCRF-GFP/^f*Grin1*+^ infused mouse is shown. RTPCR conducted on f*Grin1* mice that received unilateral CeA infusion of LV pCRF-Cre virus show a significant decrease in (**C**) *Grin1*, but not (**D**) *CRF* expression in the CeA. Dual fluorescent *in situ* hybridization was conducted on tissue from the CeA of f*Grin1* mice that received CeA infusion of LV pCRF-Cre virus. We show relative expression of *CRF* (**E, F**), *Grin1* (**G, H**), the overlay of *CRF* and *Grin1* (**I, J**) and Hoescht staining (**K, L**) of cell nuclei in the control (*Left* panels) and Infected (R*ight* panels) hemispheres.

To determine whether the virus infusion resulted in any anxiety differences we measured behavior in the open field test and found no significant differences in time in the open area (Figure 7A, F(1, 14) = .251, p>0.05) or in total distance traveled (Figure 7B, F(1,14) = .003, p>0.05). These findings replicate our previous findings that disruption of *Grin1* in CRF neurons does enhance fear acquisition but does not have effects on anxiety even when the manipulation is directly targeted to the CeA.

### Post Virus Infusion RTPCR

To determine whether LV-pCRF-Cre virus infusion into a f*Grin1* mouse reduced *Grin1* expression, we measured *Grin1* in LV-pCRF-Cre infused f*Grin1*+ mice using RTPCR. LV-pCRF-Cre was infused into f*Grin1*+ (N = 6) mice unilaterally to allow us to use the uninfected side as a control. After allowing time for infection mice were sacrificed and the CeA was extracted. We found that *Grin1* was significantly disrupted in the CeA of f*Grin1* mice (F (1,11) = 9.295, p<0.05) compared to the uninfected hemisphere. *CRF* was also measured in the same tissue and we found no significant difference in CRF expression after LV-pCRF-Cre virus infection compared to controls (F (1, 11) = 0.183, p>0.05). These findings demonstrate significant disruption in *Grin1* in the CeA of LV-pCRF-Cre/^f*Grin1*+^ mice without significant disruption of CRF.

## Discussion

We found that knockdown of *Grin1* in *CRF* containing neurons decreases the expression of *Grin1* in the amygdala and enhances fear memory formation and retention without effecting anxiety, activity level, pain sensitivity or novelty seeking behaviors. Previous work has shown that *Grin1* receptors in the central amygdala have a role in synaptic plasticity [17] and acquisition of fear memory [5], [18], but are not engaged during fear extinction [19]. Our work adds to these findings by demonstrating that disruption of *Grin1* in CRF neurons strongly facilitates fear memory formation and retention.

Why would disruption of *Grin1* expression in the amygdala facilitate fear memory formation when data has consistently shown that NMDA receptors are required during acquisition of fear conditioning in the amygdala [32], [33]? Work presented here finds that *Gria1* and *Creb1* are increased in the amygdala at baseline and after fear conditioning, in agreement with findings from previous studies showing that these targets increase when *Grin1* is disrupted [34]–[37]. Even in the absence of input from the NMDA receptor, the AMPA receptor can activate CREB along with other transcription factors [23], [38], [39]. Interestingly, we did measure *Gria1* and *Creb1* expression in the BNST (another CRF-enriched area) using RTPCR at baseline and after fear conditioning. We found no difference in expression of *Gria1* or *Creb1* compared to their Cre−/^f*Grin1*+^littermates, supporting the notion that the increase in *Gria1* and *Creb1* was not due to a generalized compensatory mechanism in CRF rich areas in Cre+/^f*Grin1*+^ mice. Therefore, increases in *Gria1* and *Creb1* in the amygdala may offer a mechanism supporting the enhancement of fear memory formation seen in Cre+/^f*Grin1*+^ mice.

Disruption of the gene that encodes NR1 (*Grin1*) disrupts a critical subunit of the NMDA receptor that is required for ion selectivity and agonist binding [40]. Cortical neurons lacking the NR1 subunit demonstrate resilience against the neurotoxic effects of exogenously applied glutamate when compared to cultured cells with normal NR1 expression (Tokita et al. 1996). One study showed that disruption of the second transmembrane segment of NR1 altered the Ca2+ permeability, sensitivity of the receptor to blockade by Mg2+ or an antagonist of the NMDA receptor and attenuated the inhibitory effects of Zn2+ [41]. These findings highlight the fundamental importance of the NR1 subunit to the functions of the NMDA receptor.

We first assessed the effect of disrupting *Grin1* containing CRF neurons throughout development as well as across CRF containing brain structures. We next tested whether fear conditioning would be enhanced using a more temporally and spatially limited disruption of *CRF* containing *Grin1* neurons. We infused a Cre recombinase dependent lentivirus driven by a CRF promoter into the CeA of floxed *Grin1* mice prior to fear conditioning. In agreement with our initial findings, we found that virally mediated disruption of *Grin1* selectively in CeA CRF neurons resulted in enhanced consolidation and retention of fear memory and delayed fear extinction. Manipulation of CRF containing *Grin1* neurons did not affect anxiety-like behavior. This finding strongly suggests that glutamatergic modulation of CRF neurons within the CeA underlies the changes in fear acquisition and extinction.

We chose to target the CeA because of its importance to fear memory formation [5] and extinction [42], as well as its high levels of CRF expression [43]. Pharmacological disruption of the CeL during fear conditioning has been shown to impair learning [5], [18], [44]. Recent studies indicate fear conditioning changes the activity of CeA neurons in a CS-dependent manner [44]–[46], suggesting learning related changes are occurring within the CeA. Data further indicates that the CeA may store fear memory in series with the lateral amygdala, providing redundancy in fear memory localization [47], [48].

One key feature of PTSD is that a mild stressor leads to an exaggerated fear reaction that is more appropriate to the level of fear expressed during the original traumatizing event rather than for the current conditions. One of our key findings is that enhanced fear learning results from disrupted *Grin1* in CRF containing cells. However, differences in fear behavior may reflect hyper-responsiveness to the CS – rather than enhanced fear learning *per se* [49], [50]. We believe enhanced fear learning is a more likely explanation for our data for the following reasons. First, there is no significant difference in freezing behavior during training until the 3^rd^ tone – shock presentation in our fear conditioning experiments. This indicates to us that the behavior of the transgenic Cre+/^f*Grin1*+^ animal and the virally infused LV-pCRF_3.0_-Cre/^f*Grin1*+^ mice are on par with their wild type and control virus infused littermate controls, respectively. Second, during the baseline of extinction training and testing there is no difference in freezing between groups, suggesting an equivalent level of fear behavior prior to stimulation. Third, the targets increased after fear conditioning in Cre+/^f*Grin1*+^ mice (*Creb1* and *Gria1*) are well characterized as involved in fear memory formation [51], [52].

Importantly, a key feature of fear-related disorders like PTSD is a marked enhancement in fear memory and resistance to fear extinction [4].

While our findings regarding the importance of fear acquisition and retention are consistent regardless of whether we test animals with transgenic disruption of NMDA1 receptor subunits within the CRF neuron population (Cre+*/^fGrin1^* ) or virus infusion of Cre recombinase targeted to CRF containing neurons (LV-pCRF_3.0_-Cre/*^fGrin1+^*). Our results regarding retention of fear extinction are less clear. Cre+*/^fGrin1+^* mice do not show a significant difference in freezing behavior during the extinction test. In contrast, mice that receive LV-pCRF_3.0_-Cre/*^fGrin1+^* virus infusion do show significantly disrupt freezing behavior during the fear extinction test. One explanation may be found in the more conservative extinction training protocol administered to LV-pCRF_3.0_-Cre/*^fGrin1+^* virus infused mice (15 trials) compared to 30 trials administered to transgenic mice. The more rigorous extinction training experience conducted with Cre+*/^fGrin1+^* mice may be sufficient to override their resistance to extinction.

We also measured *CRF* expression at baseline in the amygdala and found a noticeable, but not significant, decrease in Cre+/^f*Grin1*+^ compared to Cre−/^f*Grin1*+^ mice. In previous work [26], we found that disruption of *GABA(A)α1* in *CRF* containing neurons significantly increased *CRF* expression. Interestingly disruption of *Grin1* in CRF neurons trends towards decreasing *CRF* expression. These studies may indicate manipulation of GABAergic or glutamatergic input onto CRF neurons also modulates *CRF* expression highlighting the tight regulation of CRF by its excitatory (glutamatergic) or inhibitory (GABAergic) receptor input and warrants further study.

In humans, CRF levels are found to be correlated with severity of PTSD symptoms [11], [12]. The present work was designed to further study mechanisms underlying the contributions of CRF containing neurons to fear and anxiety disorders. Crossing a CRF-Cre mouse with a floxed *Grin1* mouse allowed for targeted disruption of *Grin1* in CRF containing neurons, decreasing *Grin1* dependent excitatory input onto this subpopulation of CRF containing neurons. Utilizing this model of subtype-specific deletion within CRF-containing neurons resulted in a very specific behavioral phenotype, that of enhanced fear acquisition and retention resulting in disrupted fear extinction. This phenotype is similar to the hallmark symptoms of PTSD wherein the initial trauma may be “over-consolidated” and difficult to extinguish. Further, we found that CeA targeting of CRF- *Grin1* neurons results in an identical enhancement of auditory fear conditioning and an even stronger disruption of extinction. These findings highlight the critical role of CeA *Grin1* modulation of CRF neurons in fear memory formation and inhibition.

Previous work from our lab shows disruption of *GABA(A)α1* selectively in CRF neurons increases anxiety, has no effect on fear conditioning and disrupts fear extinction [26]. Our present work shows that disruption of *Grin1* in CRF containing neurons has no effects on anxiety but strongly enhances fear acquisition and retention. Even though we are manipulating a relatively small subset of neurons, we measure strikingly significant changes in fear and anxiety behaviors across these different manipulations of CRFergic neurons. These findings highlight the critical contribution of the CRF neuronal population to modulation of fear and anxiety behavior. Increased understanding of the differential contributions of subtypes of CRF neurons will be of critical importance in moving towards development of treatments targeting fear and anxiety disorders.

## Supporting Information

Data S1 The raw data from the **(A)** baseline RTPCR experiments showing fold change data from Cre−/^f*Grin1*+^ and Cre+/^f*Grin1*+^ mice. **(B)** Baseline anxiety tests for Cre+/^f*Grin1*+^ and Cre−/^f*Grin1*+^ showing open field (time in center zone, distance traveled), plus maze (time in open arm, distance traveled) and shock reactivity (amplitude) data. (**C**) Raw fold change data from post fear conditioning RTPCR for Cre−/^f*Grin1*+^ and Cre+/^f*Grin1*+^ mice is shown for amygdala (Gria1, Creb1) and BNST (Gria1, Creb1 and Grin1). (**D**) Raw data from auditory fear conditioning of Cre−/^f*Grin1*+^ and Cre+/^f*Grin1*+^ mice is shown at baseline and over the 5 tone- shock pairings. Extinction training (**E**) and extinction testing (**F**) freezing data are shown for Cre−/^f*Grin1*+^ and Cre+/^f*Grin1*+^ mice including baseline. Data during tone presentation is grouped into 3 trial bins. (**G**) Raw data from auditory fear conditioning of LV pCRF-Cre/^f*Grin1*+^ and LV pCRF-GFP/^f*Grin1*+^ mice is shown at baseline and over the 5 tone- shock pairings. Extinction training (**H**) and extinction testing (**I**) freezing data are shown for LV pCRF-Cre/^f*Grin1*+^ and LV pCRF-GFP/^f*Grin1*+^ mice including baseline. Data during tone presentation is grouped into 3 trial bins.
(XLSX)
